# The State-of-the-Art of Gene Editing and its Application to Viral Infections and Diseases Including COVID-19

**DOI:** 10.3389/fcimb.2022.869889

**Published:** 2022-06-09

**Authors:** Yousef M. Hawsawi, Anwar Shams, Abdulrahman Theyab, Jumana Siddiqui, Mawada Barnawee, Wed A. Abdali, Nada A. Marghalani, Nada H. Alshelali, Rawan Al-Sayed, Othman Alzahrani, Alanoud Alqahtani, Abdulrahman M. Alsulaiman

**Affiliations:** ^1^ Research Center, King Faisal Specialist Hospital and Research Center, Jeddah, Saudi Arabia; ^2^ College of Medicine, Al-Faisal University, Riyadh, Saudi Arabia; ^3^ Department of Pharmacology, College of Medicine, Taif University, Mecca, Saudi Arabia; ^4^ Department of Laboratory & Blood Bank, Security Forces Hospital, Mecca, Saudi Arabia; ^5^ Department of Biology, Faculty of Science, University of Tabuk, Tabuk, Saudi Arabia; ^6^ Genome and Biotechnology Unit, Faculty of Science, University of Tabuk, Tabuk, Saudi Arabia; ^7^ Bristol Medical School, Faculty of Health Sciences, University of Bristol, Bristol, United Kingdom; ^8^ Department of Medical Microbiology, Prince Sultan Military Medical City, Riyadh, Saudi Arabia

**Keywords:** gene editing, COVID-19, CRISPR/Cas9, vaccines, viral infection

## Abstract

Gene therapy delivers a promising hope to cure many diseases and defects. The discovery of gene-editing technology fueled the world with valuable tools that have been employed in various domains of science, medicine, and biotechnology. Multiple means of gene editing have been established, including CRISPR/Cas, ZFNs, and TALENs. These strategies are believed to help understand the biological mechanisms of disease progression. Severe acute respiratory syndrome coronavirus 2 (SARS-CoV-2) has been designated the causative virus for coronavirus disease 2019 (COVID-19) that emerged at the end of 2019. This viral infection is a highly pathogenic and transmissible disease that caused a public health pandemic. As gene editing tools have shown great success in multiple scientific and medical areas, they could eventually contribute to discovering novel therapeutic and diagnostic strategies to battle the COVID-19 pandemic disease. This review aims to briefly highlight the history and some of the recent advancements of gene editing technologies. After that, we will describe various biological features of the CRISPR-Cas9 system and its diverse implications in treating different infectious diseases, both viral and non-viral. Finally, we will present current and future advancements in combating COVID-19 with a potential contribution of the CRISPR system as an antiviral modality in this battle.

## 1 Introduction

Applying desired modifications to genomes (i.e., genome editing) has been a craved ambition in molecular biology, medicine, and biotechnology. Indeed, the ability for precise editing of DNA bases bears a tremendous value for answering countless questions concerned about human genomes. Therefore, marvellous efforts have been employed to investigate restriction enzymes, which were first discovered in bacteria, and advance the field of recombinant DNA technology (Adli, 2018). Gene editing is an emerging technology that aims to introduce targeted modifications into the genome using nucleases engineered to cut a specific genomic target sequence (Shim et al., 2017). Programmable nucleases induce double-strand breaks (DSBs) at a particular site in the genome (Rouet et al., 1994), leading to activation of cellular DNA repair mechanisms and thus facilitating the introduction of targeted genomic modifications at the specific areas of interest (Rouet et al., 1994). This process occurs through either of the two fundamental repair mechanisms: homology-directed repair (HDR) and the non-homologous end joining (NHEJ) (O'Driscoll and Jeggo, 2006). HDR removes DSBs by utilizing a donor homologues DNA segment on the same or different DNA molecules, resulting in the introduction of the anticipated sequence changes. NHEJ removes DSBs from the genome by nonhomologous end-joining (through insertions and deletions) without homology requirements, leading to a gene inactivation (Iliakis et al., 2004). Three powerful classes of site-specific DSB nucleases have been established to facilitate genome editing. These are (1) zinc finger nucleases (ZFNs), (2) transcription activator-like effector nucleases (TALENs) and (3) and the clustered regularly interspaced short palindromic repeats (CRISPR) system (Carroll, 2014). These tools have been successfully utilized in targeting, deleting, and replacing mutations within the gene, making them versatile tools for gene therapy. This review offers a concise history of different technologies used in gene editing. It highlights some of the recent advancements, mainly CRISPR technologies in viral infectious diseases, focusing on its possible applications in combating COVID-19 disease.

## 2 History of Gene Editing

Although the concept of introducing new genetic elements into an organism had begun in the 1970s (Esvelt and Wang, 2013), in recent years, the development and applications of gene editing methodologies have dramatically accelerated. This has enabled scientists to manipulate genes in various diseases (Rothstein, 1983). The process of the HDR technique, which is conducted through homologous recombination by exchanging the sequence of nucleotides between two analogous or identical double-stranded molecules (Iliakis et al., 2004), was first implemented in yeasts in the 1970s of the 20th Century (Rothstein, 1983). In 1981, the first transgenic animal was generated by transferring the gene of a rabbit into the mouse genome (Costantini and Lacy, 1981). NHEJ, on the other hand, which knockout genes by nonhomologous end joining, was first described by Moore and Haber in 1996 (Bibikova et al., 2002; Hefferin and Tomkinson, 2005). Three forms of engineered nucleases have been utilized to trigger DNA cleavage to generate DSBs, which are eventually subjected to repair through any of the two possible cellular repair mechanisms (i.e., NHEJ or HDR). The first programmable nuclease developed for gene editing was ZFN. The second nuclease, TALEN, was first discovered in 1989 in a group of gram-negative bacterial plant pathogens (Bonas et al., 1989). In 2007, TALEN’s capability to bind to DNA was described by Romer et al. (2007), and in 2009, the DNA binding code for TALE’s proteins was deciphered (Moscou and Bogdanove, 2009; Boch et al., 2009).

The third engineered nuclease is the clustered regularly interspaced short palindromic repeats (CRISPR). CRISPR was initially described by Ishino et al. in 1987 when they discovered an unusual repetitive DNA sequence in the *E. coli* genome (Ishino et al., 1987), which was subsequently identified in substantial proportions of other bacterial and archaeal genomes. In a separate study, Francisco Mojica elucidated the CRISPR locus by reporting the structures and characteristics of these short palindromic repeats in 2000 (Mojica et al., 2000). Over the next five years, Mojica continued to look deeper into these repeats and discovered that some of the sequences of CRISPR acquired homology with bacteriophage genome, indicating a defensive mechanism of these repeats against phages (Mojica et al., 2005). Later, Ruud Jansen and colleagues found that encoding of some CRISPR-associated (Cas) proteins takes place adjacent to the repeat clusters mediating the viral sequence capturing (Jansen et al., 2002). In the following year, scientists began to uncover the mechanisms of CRISPR-Cas systems with invader phage. In 2008, the first portion of the puzzle was discovered by John van der Oost et al., who demonstrated the existence of CRISPR RNAs (crRNAs) spacer sequences in the genome of *E. coli*. These spacers are created using the viral DNA and are transcribed into small RNAs, known as crRNAs. These crRNAs can target the DNA after activating the Cas protein (Brouns et al., 2008). In 2011, Emmanuelle Charpentier’s group discovered a second small RNA in *Streptococcus pyogenes*, trans-acting RNA (tracrRNA). They showed that tracrRNA generates a duplex with crRNA, directing Cas9 to its specific targets (Deltcheva et al., 2011). In 2013, the earliest CRISPR application of CRISPR-based editing in eukaryotic cells was announced by targeting human and mouse cells (Cong et al., 2013; Mali et al., 2013). Indeed, CRISPR is considered the dominant genome-editing method worldwide; however, ZFNs and TALENs are still in use (Lee et al., 2018).

## 3 Genome Editing Platforms

### 3.1 Zinc Finger Nucleases

ZFNs were used as engineered DNA-binding proteins in various organisms, including human cells (Hauschild-Quintern et al., 2013). The ZFNs design allows for a targeted introduction of a DSB into a specific target locus. It stimulates gene targeting 100- to 10,000-fold through the activation of DNA repair mechanisms in case the donor DNA is absent (Zhang et al., 2016; Saha et al., 2019). ZFNs consist of an engineered Cys2-His2 ZF domain infusion with the nuclease domain of the type IIS restriction enzyme FokI (Gaj et al., 2013). An individual zinc finger contains about thirty amino acids in a preserved ββα configuration (Gaj et al., 2013). A pair of ZFN subunits are usually designed to recognize the targeted sequence in a tail-to-tail conformation. The invention of zinc finger nickases (ZFNickases), in which induction of site-specific single-strand breaks in genomic DNA results in HDR, advanced the ZFN-mediated genome editing specificity (Wang et al., 2012). The ZFN pair can be bound after translation to its specific target, and then DNA binding occurs, resulting in DNA cleavage after dimer *FokI* catalytic domains (Hauschild-Quintern et al., 2013). ZFN expression has proven effective in human cells when incubated with transfected cells for a few days at 30°C. The possible explanations for stress-induced cells and hypothermia are protein degradation and decreased mRNA, which might protect the modified cells (Hauschild-Quintern et al., 2013). Therefore, a site-specific DSB can be prompted by a ZFN pair at the specific site of the target molecule.

For current therapeutic applications in gene therapy, a promising area of consideration is dealing with the induction of pluripotent stem cells (iPS) using patient-specific embryonic stem cells (ESCs) to prevent the chronic rejection of transplants (Hauschild-Quintern et al., 2013). ZFNs have unique features; because of their compact sizes, they can access every genome compartment, thus making them suitable to be used in the gene therapy (David et al., 2019). Another criterion of ZFNs is that they can help DNA binding distinguish an epigenetic modification within a given target (Choo, 1998). Gene-driven targeting by ZFNs in laboratory mice was primarily achieved by mRNA or DNA microinjection into fertilized oocytes (Cui et al., 2011; Chen et al., 2014). The founder animals can transmit the modified genes to their offspring through their germline (Hauschild-Quintern et al., 2013). By injecting ZFN with the donor template into pronuclear DSB, it can be introduced into pseudopregnant females, who require screening and breeding to produce offspring with the modified genotype. ZFNs can cause a different mutation in different cell types by cleaving the DNA multiple times (Meyer et al., 2010).

### 3.2 Transcription Activator-Like Effector Nucleases

The DNA-binding domain of TALE contains 10-30 tandem repeats or monomers of 34 amino acid residues. These residues are exceptionally preserved except for the -position 12 and 13- hypervariable amino acid residues called repeat-variable di-residue (RVD). These RVDs are responsible for recognizing a specific nucleotide (Nemudryi et al., 2014). Thymine is the first base recognized by an N-terminal region of TALE; other commands are subsequently bound to RVD (Nemudryi et al., 2014). Like ZFNs, TALEN is also generated by fusing the engineered TALE-binding domain with the FokI endonuclease element (Lee et al., 2018). Applying the TALEN technique would greatly aid the generation of knockout mice.

The first knockout mice were described in 2013. The FokI-based engineered endonuclease is highly effective in inducing mutations in the target gene (Sung et al., 2013), and successful germline transmission is established based on knockout mice (Qiu et al., 2013). In each gene, one or more founders were backcrossed to wild-type mice to determine if a TALEN-promoted mutation has the option to pass through the germline efficiently (Bortesi and Fischer, 2015). The next generation was born successfully and inherited at least one mutant allele from the founders (Qiu et al., 2013). Successful germline transmission was detected in every founder through test breeding. Thus, TALEN-induced gene mutation was heritable in genetically different mice (Qiu et al., 2013). *In vitro* injection of mRNA to each TALEN pair was transcribed into the cytoplasm of one-cell mouse embryos from the successful generation of mice in which the target gene was disrupted. After being injected, the embryos followed either an overnight culture or were moved to pseudopregnant females. The pip genomic DNA was extracted from each embryo injected with TALEN, followed by PCR amplification of the target site (Qiu et al., 2013).

### 3.3 Clustered Regularly Interspaced Short Palindromic Repeats

#### 3.3.1 CRISPR Systems Structure and Origin

CRISPR and CRISPR-associated protein nine nuclease (Cas9) is synthesized from *Streptococcus pyogenes.* It is historically a defence surveillance mechanism provided by prokaryotic adaptive immunity to protect bacteria against foreign DNA such as viral invasion (Xiao et al., 2013; Bortesi and Fischer, 2015). The Cas endonuclease proteins have a cutting activity to cleave the foreign nucleic acids into fragments, thus destroying the attackers (Cong et al., 2013). The Cas9 nuclease creates a double-stranded break in DNA at a site outlined by a short guide RNA. In the type II CRISPR/Cas system, short foreign DNA segments named “spacers” are subjected to integration within the CRISPR genomic loci. Then, spacers are transcribed and processed into short CRISPR RNAs (crRNAs) that use Cas proteins to initiate cleavage and silencing sequence-specific pathogenic DNA (Sternberg et al., 2016). Another study showed that recognizing the target by the Cas9 protein needs a seed sequence inside the crRNA and a preserved sequence for dinucleotide-containing protospacer adjacent motif (PAM) located upstream of the crRNA-binding region (Gaj et al., 2013). CRISPR/Cas9 system can be classified into two main categories, class 1 and 2, and further classified into six and 48 sub-types. Class 1 CRISPR-Cas9 encompasses about 90% of all CRISPR loci and is mainly found in fungal genomes. On the other hand, class 2 CRISPR-Cas9 accounts for only 10% and is present mainly in the bacterial genome compared to other organisms (Katalani et al., 2020).

#### 3.3.2 CRISPR/Cas Systems at a Glance

**Figure 1:**
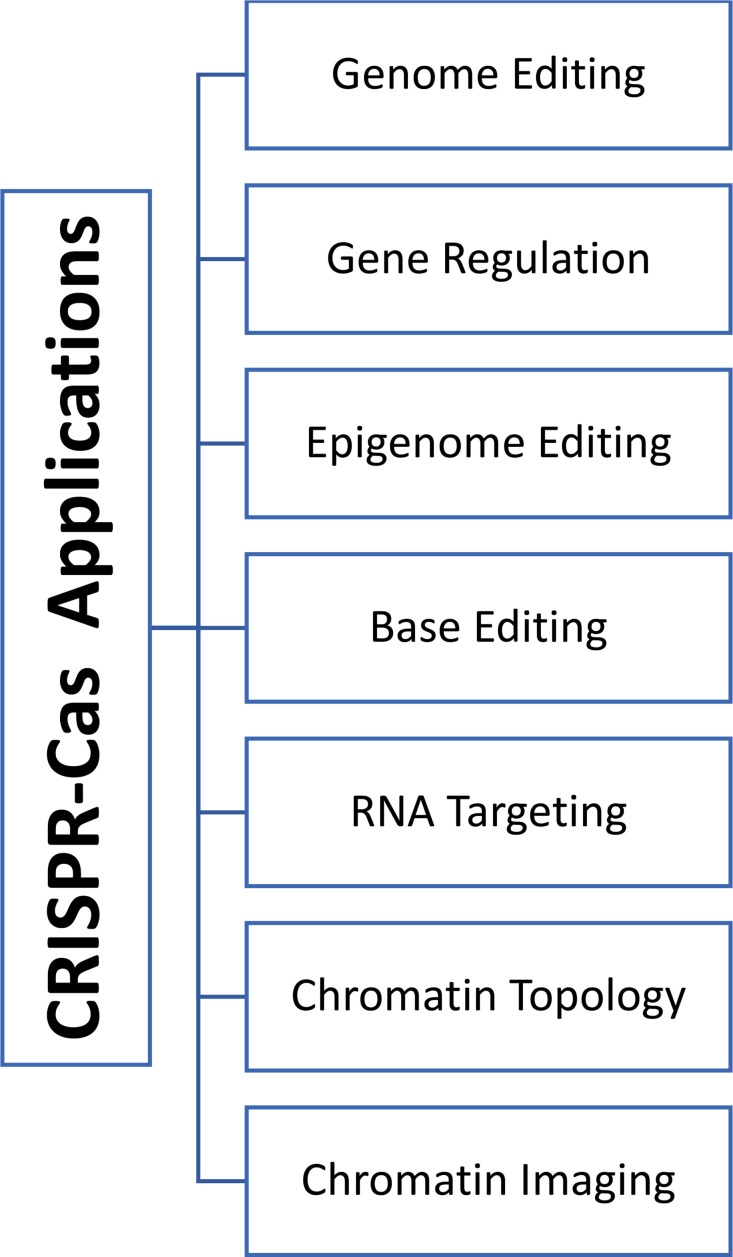
Different areas of applications of CRISPR-Cas technologies.

Emmanuelle Charpentier and Jennifer Doudna were awarded the 2020 Nobel Prize in chemistry for affording CRISPR/Cas technology to correct the genomic DNA (Derry, 2021). Previously gene therapy was based on adding functional alleles to correct the underlying disorder for so-called gene augmentation therapies. Yet this approach is limited by the size of the affected genes that might exceed the capacity of the viral vectors causing difficulty in introducing the full-length alleles. Moreover, the gene augmentation approach cannot fix disorders that result from genetic mutations (gain-of-function mutations or dominant-negative alleles). On the contrary, the CRISPR-Cas9 system can correct both types of mutations, whether they are loss or gain of function mutations, regardless of the size of the targeted genes (Xu CL et al., 2019). CRISPR/Cas9 was first introduced into mammalian cells in 2013, offering many advantages over the other traditional genome editing types of machinery, ZFN and TALEN. Using the CRISPR/Cas9 system allows for designing a complementary sgRNA maintaining the same Cas9 nuclease in all cases. Secondly, CRISPR/Cas9 technology is characterized by multiplexing properties that simultaneously target various genomic loci by designing multiple sgRNAs (Xiao-Jie et al., 2015). For example, in agricultural areas, scientists were able to use CRISPR/Cas9 to knock out (KO) and knock-in (KI) a gene at the same locus (KO-MSTN and KI-PUFA genes in gouts), resulting in animals with high muscle mass and with increased resistance to cardiovascular disorders (Zhang et al., 2018). Several criteria such as efficiency, simplicity, robustness, cost-effectiveness, and flexibility to use paved the way for CRISPR/Cas9 systems to be adopted as a method of choice for gene editing reasons (Adli, 2018). Therefore, CRISPR/Cas9 technology has exploded at a breathtaking pace and has revolutionized different areas, including gene modulations, epigenetic editing, chromatin imaging, and engineering in the laboratory and preclinical settings in many creatures (**Figure 1**) (Jinek et al., 2012; Xu CL et al., 2019). The field of CRISPR technology continued to advance as second-generation genome editing tools have developed. Unlike the standard Cas9 that causes DSBs and indels at the affected site, these editor tools enable the direct conversion between different bases (e.g., C into T) without cutting the DNA (nickase Cas9) (Gaudelli et al., 2017). This conversion ability results in early STOP codons in genes; therefore, the CRISPR-STOP tool is a practical and less detrimental approach than the wild-type Cas knockout systems (Billon et al., 2017).

#### 3.3.3 CRISPR/Cas System Obstacles and Side Effects

**Figure 2:**
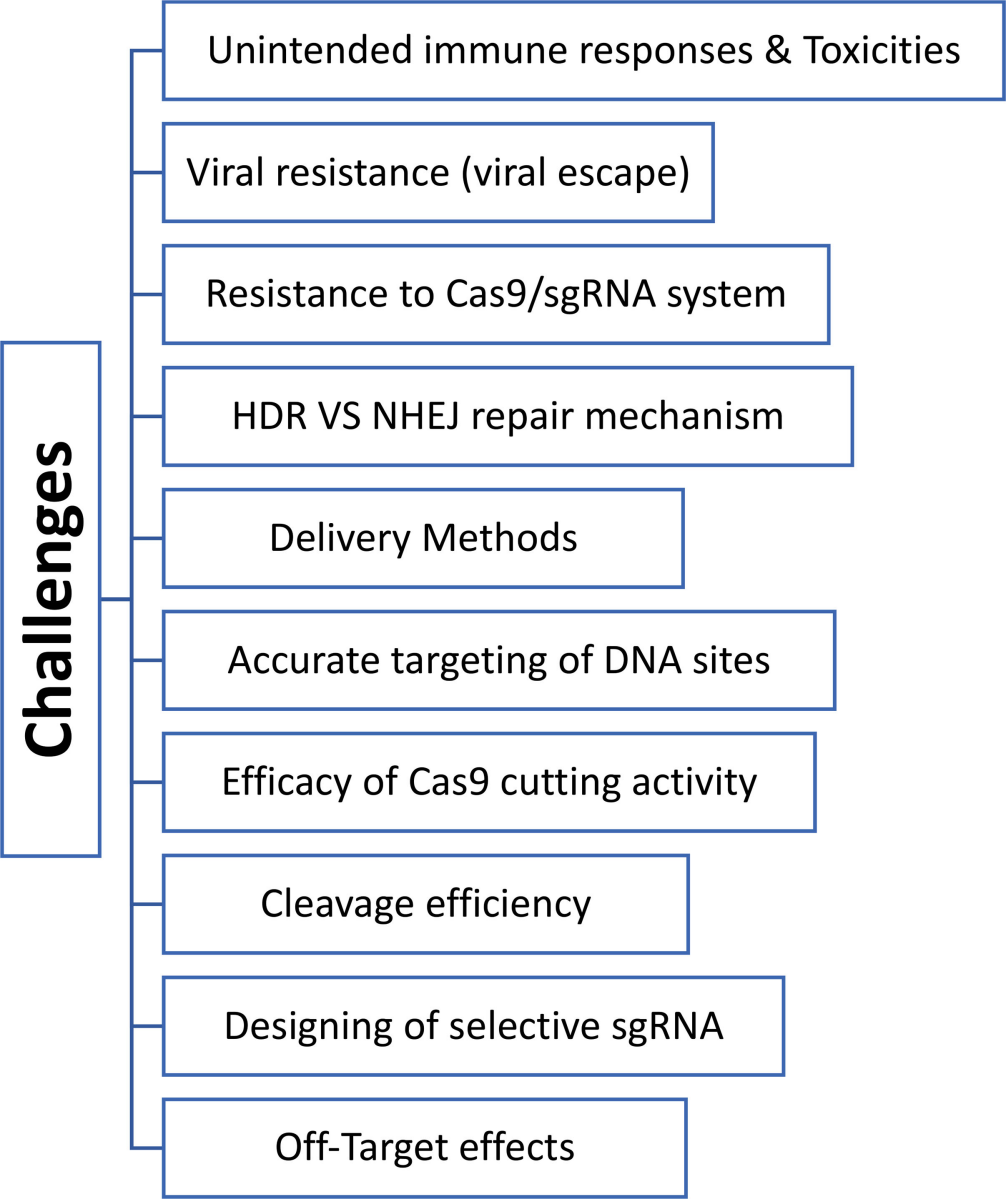
Diagram shows various obstacles and challenges of CRISPR-Cas technologies in treating different diseases.

While CRISPR technology has displayed numerous promises in genome editing, many side effects are linked to this system and must be profiled and assessed correctly. These unwanted effects include accurate targeting of DNA site, off-target results, designing of selective sgRNA, the efficacy of Cas9 cutting activity, the occurrence of HDR vs NHEJ repair mechanisms, viral escape (acquisition of mutation), resistance to Cas9/sgRNA system, and the type of delivery tool, (**Figure 2**) (Xiao-Jie et al., 2015; Lino et al., 2018). Off-target effects of CRISPR/Cas9 comprise the principal barrier in conducting a clinical trial of the CRISPR/Cas system. This must be addressed vigilantly when applied to *in vivo* studies to prove the safety profile of the viral vectors (Kim et al., 2015; Lin et al., 2021). Other crucial obstacles for *in vivo* applications are the specificity and accuracy of the editing process and the delivery method of the CRISPR/Cas9 system. In this respect, the early two clinical trials were conducted in France and the UK of children with X-linked severe combined immunodeficiency (SCID X-1). Five children out of 20 developed T-cell leukemia with one refractory case as the delivered genetic material was not added to the genome (Check, 2002; Kaiser, 2003; Thomas et al., 2003). Another misfortune case of an 18-year-old male with ornithine transcarbamylase (OTC) deceased four hours post-treatment with a genetic cargo delivery tool that caused severe inflammatory responses (NIH, 2002; Marshall, 1999). Both tragedies emphasize the importance of developing site-specific gene-editing machinery and introducing a safe and efficient transport vehicle which are the crux of gene therapy technologies (Xiao-Jie et al., 2015; Lino et al., 2018). Indeed, several delivery vehicles have been developed that include physical delivery (such as microinjection and electroporation), viral vectors (adeno-associated virus (AAV), and full-sized adenovirus and lentivirus), and non-viral vectors like lipid nanoparticles, cell-penetrating peptides (CPPs), DNA ‘nanoclews’, and gold nanoparticles (Lino et al., 2018).

## 4 Applications of CRISPR/Cas Technology in Targeting Viral and Nonviral Infectious Diseases

Gene editing is a promising avenue in treating various human diseases, including infectious and non-infectious disorders such as cancer, diabetes, heart failure, hematological diseases, and neurodegenerative conditions. Over 2000 clinical trials have been conducted with the main focus on genome editing of the primary defects of the immune system, viral infections, and cardiovascular diseases (Li et al., 2020). However, only a few gene therapeutic compounds have been recently approved, such as Gendicine in China for squamous cell carcinoma of the head and neck. The other one is Cepero in Europe for brain cancers (Wirth et al., 2013; Allen and Feigin, 2014; Braunwald, 2015). Because CRISPR/Cas9 was initially identified as a defence mechanism in bacteria and archaea, its employment in manipulating different diseases has been suggested. Furthermore, CRISPR/Cas also showed the ability to modify both DNA and RNA sequences, thus enabling the nucleases to modulate both the viral and the host genomes (Escalona-Noguero et al., 2021). The nucleases also possess the practical ability to edit multiple genes concurrently. Using CRISPR/Cas reduces the need for engineering, choosing and verifying the target proteins (Janusz et al., 2020). It also uses small RNA that is costless, highly specific and versatile, and easy to design. These make CRISPR/Cas superior to other gene-editing modalities as a therapeutic approach for viral diseases (White et al., 2015; Najafi et al., 2022).

### 4.1 Implication of CRISPR Technology in Targeting Viral Infections

**Figure 3:**
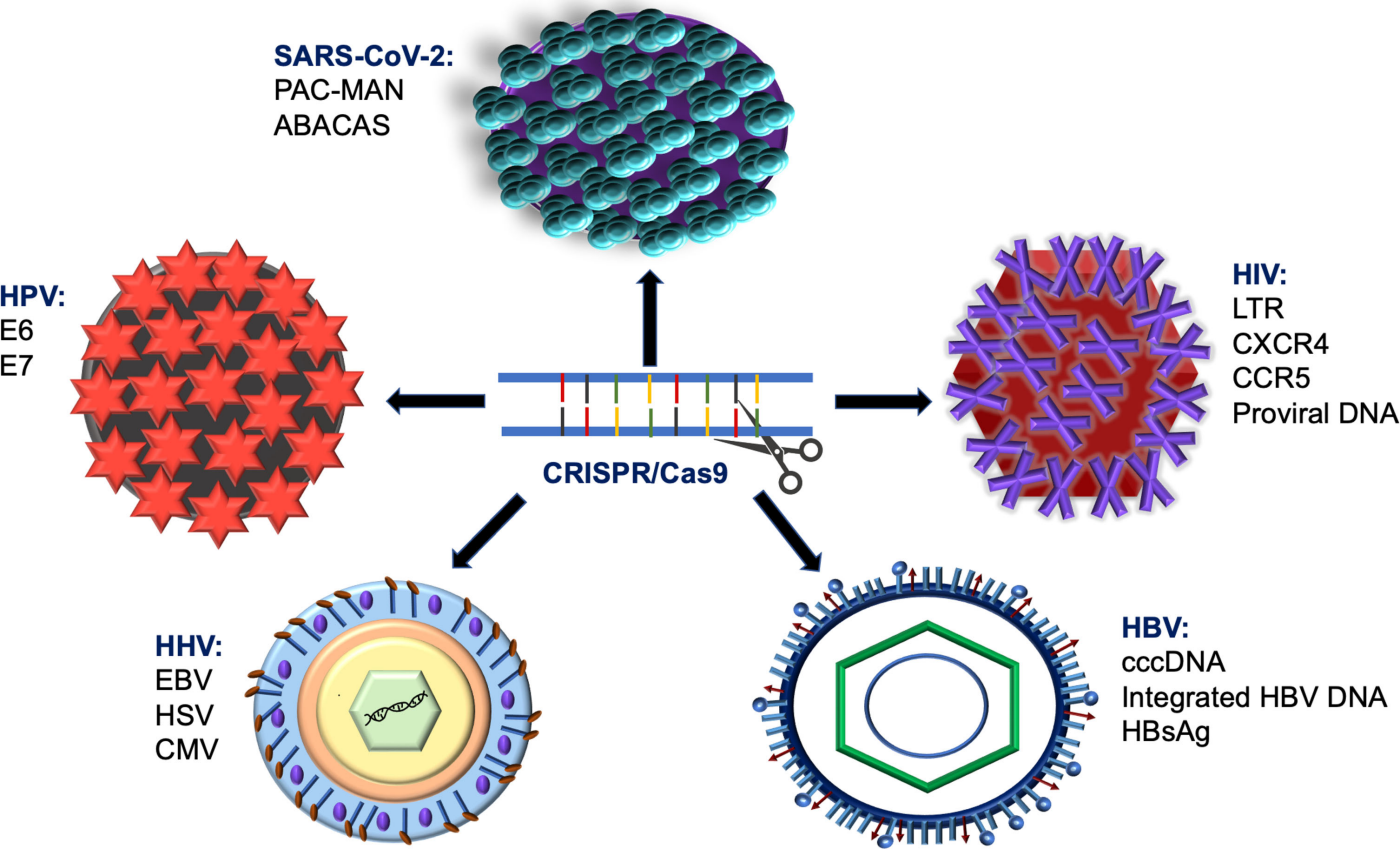
Shematic diagram describes using of CRISPR/Cas9 system in targeting various types of viral infections.

Among other gene-editing technologies, CRISPR/Cas9 was found to deliver new competencies in fighting against human infectious virus illnesses that convey a substantial risk to human health (Moens, 2018) and increase the socioeconomic load on the public health organizations globally (Doerflinger et al., 2017). Herein, we will discuss the anti-viral approach of CRISPR/Cas9 to manage the infectious potency of severe human viruses, including the human immunodeficiency virus type 1(HIV-1) and type 2 (HIV-2), Hepatitis B viral infection (HBV), human papillomaviruses (HPV), and other human herpesviruses, (**Figure 3**). The first three groups of viruses displayed more risk hazardous to the patients and featured the ability to form dormant reservoirs in the affected cells and overuse cellular nutrients. Therefore, the clinical challenges in curing these viruses are frequently encountered, and the therapeutic success is relatively low in contrast to herpesviruses (White et al., 2015).

#### 4.1.1 Human Immunodeficiency Viruses 1 and 2

The two lentiviruses, HIV-1 and HIV-2, belong to the same family of Retroviridae and subfamily of Orthoretrovirinae (2016); both types share several features related to genomic structure, ways of transmission, and clinical outcomes (Clavel et al., 1986). HIV-1 infection spreads globally, and the infected individuals usually present with progressive disease. On the other hand, HIV-2 is mainly limited to West Africa. The infected patients showed more protective immune responses against the virus that restricted the virus progression and reduced the need for antiretroviral therapy (Nyamweya et al., 2013). Transmission of HIV takes place through three common routes: (1) sexual intercourse, (2) direct injections with HIV-contaminated products, and (3) vertical transmission from an infected mother to her fetus.

Furthermore, HIV-infected patients undergo three distinct phases: acute HIV infection (seroconversion), asymptomatic stage, and the chronic condition of Acquired Immune Deficiency Syndrome (AIDS) (Huang et al., 2012). AIDS is a consequence of persistent infection by either of two lentiviruses: HIV-1 and HIV-2, and is considered a public health crisis that affected 38 million people worldwide in 2019 (UNAIDS, 2019). The first study that described the efficiency of the CRISPR/Cas9 system in suppressing HIV infection was conducted by Ebina et al. (2013). In this report, CRISPR/Cas9 mutated the long terminal repeat (LTR) of HIV-1, causing a dramatic reduction in the viral expression load *in vitro*. Additionally, subsequent studies targeting either HIV gens or LTR sequence of HIV-1 DNA have demonstrated similar suppression results (Ebina et al., 2013; Hu et al., 2014; Christian et al., 2022). Moreover, the individual research reported another approach to battel HIV infection through targeting the cell surface receptors CXCR4 on the host cells making the cells resistant to viral entry and subsequent infection (Hou et al., 2015).

One of the significant challenges in treating HIV infection is eradicating the latent HIV dormant infected cells, i.e., targeting the integrated proviral DNA. Bialek et al. used the CRISPR/Cas9 system to activate the inert viral particles in their cellular reservoirs, thus making them susceptible to antiviral therapy (Bialek et al., 2016). Likewise, dual treatment with the two strategies, including CRISPR/Cas9 and LASER-ART (sequential long-acting slow-effective release antiviral therapy), has displayed fruitful results in depletion of the latent HIV-1 infection *in vivo* models. Indeed, the infected humanized mice treated with this modality showed no virus detection in blood, brain, bone marrow, or lymphoid tissues. Interestingly, no off-target effects linked to CRISPR systems, which is a part of this dual therapeutic modality, were detected (Dash et al., 2019). Other places for HIV reservoirs are located in astrocytes, microglial cells, and macrophages in the brain (Fischer-Smith et al., 2001). HIV/AIDS patients with brain involvement presented with impairment in many neurological and behavioural cognitive functions due to neurotoxic substances produced by HIV-infected cells in the brain (Heaton et al., 2010). The major obstacle in treating NeuroAIDS patients is crossing the blood-brain barrier to target the HIV dormant/latent cells, such as astrocytes, within the brain tissue. Using the CRISPR/Cas9 tool, Huang et al. designed novel guide RNAs (gRNAs) to target the HIV provirus in HIV latent astrocytes resulting in significant eradication of virus particles *in vitro* (Huang and Nair, 2017).

The CRISPR/Cas9 strategy showed the ability to inhibit HIV viral replication; it was also reported to facilitate the generation of viral resistance or viral escape phenomena (Wang et al., 2016). Cas9, through single guide RNA (sgRNA), induces the cleavage site of the HIV-1 genome that is repaired by NHEJ machinery, causing insertions and deletions (indels) (Ran et al., 2013). Nevertheless, these indels are lethal for the virus; some of these mutations generated more competent replicating viruses (CRISPR-Cas9-resistant HIV-1 strains) resistant to anti-viral therapy (Yoder and Bundschuh, 2016; Lee, 2019). To overcome the viral escape problem, the CRISPR/Cas9 strategy has been modified to use two or more gRNAs to target the conserved HIV-1 motif, the combinatorial CRISPR-Cas9 approach. Applying this modality has demonstrated effective suppression of HIV replications and further prevention of viral escape or viral resistance problem (Lebbink et al., 2017; Najafi et al., 2022). The delivery of the engineered Cas9/multiplexed-sgRNA system into the human cells HEK293 has shown successful results in abolishing the intracellular various or parvoviral genome compared to an individual targeting Cas9 (Liao et al., 2015). Nevertheless, Cas9/multiplexed-sgRNA system has grown targeting the two coreceptor genes such as CCR5 and CXCR4 has not yet been accomplished (Lin et al., 2021).

In 2019, researchers from China declared a successful treatment of HIV-1 patients with acute lymphocytic leukemia. This clinical study performed bone marrow transplantation with CCR5-depleted hematopoietic progenitors’ cells. The patient’s cells were harvested and ex-vivo edited using CRISPR therapy, then reintroduced into the patient (Xu L et al., 2019). A subsequent study authenticated that disruption of CCR5 expression is a promising tool in treating HIV patients. Targeting CCR5 expression *via* CRISPR/Cas9 mediated HDR in four cell lines, including freshly healthy human cells, dramatically suppressed HIV-1 replication. Indeed, the generated cells that are highly deficient in CCR5 showed resistance to HIV-1 infection (Scheller et al., 2022).

The origin of the CRISPR system as a self-defensive mechanism in bacteria against foreign DNAs and viruses allowed its contribution as a therapeutic tool in combating different viral and non-viral diseases. The story of vaccination has gained renown as it saves millions of people from various infectious pathogens. However, the interindividual variability in response to vaccines and sufficient immunity and neutralizing antibodies remains a challenge in designing vaccines against some viruses, including HIV, thus requiring further investigations (Doerflinger et al., 2017). Accumulating studies have been conducted to overcome the barriers associated with traditional vaccination by applying CRISPR-based strategies (Binnie et al., 2021). Using CRISPR/Cas system, researchers were able to modify the heavy chain locus in activated human B cells to generate HIV-1- specific neutralizing antibodies. Then, these B cells were transferred into the mice to generate an adequate amount of antibodies to suppress HIV-1 viremia and confer immunity. This strategy would eventually assist in producing vaccination against viruses and overcome the inconsistency in the immune response that accompanies the traditional vaccination methods (Hartweger et al., 2019).

#### 4.1.2 Hepatitis B Virus

HBV is a double-stranded circular DNA virus that belongs to the *Hepadnaviridae* family. The HBV genome contains four central genes, S, C, X, and P encode for surface antigen, core protein, X protein, and a DNA polymerase (McNaughton et al., 2019). HBV double-stranded DNA is converted into covalently closed cyclic DNA (cccDNA), which is required during the duration of infection to produce a template for generating mRNAs and pre-genomic RNA (Glebe and König, 2014). The chronicity of HBV infection is associated with an increased risk of developing liver cirrhosis and, consequently, hepatocellular carcinomas (HCC). Chronic inflammation is caused by the persistence of some viral particles in the hepatocellular reservoirs in the form of cccDNA (Doerflinger et al., 2017). Therefore complete eradication of chronic hepatitis B (CHB) infection necessitates efficient elimination of cccDNA of HBV, which cannot be achieved with current antiviral treatments (Emery and Feld, 2017). Thus explicating the relapsing of the HBV infection after completing the treatment (Fanning et al., 2019). Therefore, gene therapy that targets cccDNA holds a potential therapeutic avenue to attain complete clinical curing of CHB infection (Maepa et al., 2015; Bloom et al., 2018). Multiple studies have investigated the application of CRISPR/Cas9 for targeting and cleavage of many essential loci of HBV such as cccDNA, surface antigen, and X gene in cell cultures or animal models (Seeger and Sohn, 2014a; Kennedy et al., 2015; Ramanan et al., 2015a; Zhen et al., 2015; Yan et al., 2021; Martinez et al., 2022). Seeger and Shon demonstrated the first use of the CRISPR/Cas9 strategy to disrupt the HBV cccDNA. Two different mutations were generated by the CRISPR/Cas9 system in the HBV genome, a single nucleotide indel and a large mutational deletion in cccDNA. These mutations resulted in significant suppression of episomal DNA in HepG2/NTCP cells and reduction of HBV core antigen and other proteins up to 10 fold (Seeger and Sohn, 2014a).

The first validation of the CRISPR/Cas9 system using a realistic simulation model of HBV infection was demonstrated in humanized chimeric mice. Introducing the AAV2/WJ11-Cas9 construct revealed remarkable inhibition of cccDNA and HBcAg, HBsAg, and HBV DNA in hepatic tissues without notable cytotoxic effect (Kayesh et al., 2020). Accumulating evidence both *in vitro* and *in vivo* has confirmed the previous findings and showed the ability of the CRISPR/Cas9 strategy to disrupt cccDNA and inhibit the HBV replication (Zhen et al., 2015; Kennedy et al., 2015; Ramanan et al., 2015b). Furthermore, a new talented technology of CRISPR system has developed to permanently damage the HBV genome and ultimately reach a complete cure. This was achieved by causing premature STOP codons without inducing a double-strand break (DSB) of the host genome (Yang and Yang, 2022).

The presence of the cccDNA and incorporation of the viral DNA into the host genome accelerates the progression of CHB and the development of hepatocellular carcinomas (Martinez et al., 2022). The CRISPR/Cas9 system successfully abolishes cccDNA, yet the integrated HBV DNA particles remain potent pro-oncogenic mediators. In the HBV cell line, complete removal of the full-length integrated HBV DNA fragment was accomplished using the CRISPR/Cas9 strategy. Additionally, elimination of cccDNA, HBsAg, and HBeAg that last for more than ten months were also attained. The CRISPR/Cas9 strategy delivered another promising attempt in targeting the cccDNA and integrated HBV DNA yielding significant clearance of HBV core antigen and the mutated cccDNA (Seeger and Sohn, 2014b). These results indicated the potential implication of the CRISPR/Cas9 system in treating HBV patients and providing a radical cure for this illness (Li et al., 2017; Stone et al., 2021).

Chronic infection with HBV with a persistently elevated level of HBV surface antigen (HBsAg) is considered another critical risk factor for the development of HCC. Interestingly, the CRISPR/Cas9 strategy revealed a powerful effect in destroying the intrahepatic HBV genome and reducing HBsAg levels *in vitro* and in animal models (Lin et al., 2014; Chen et al., 2021). In one study, CRISPR/Cas9 was designed with specific single-guide RNAs (sgRNAs) to knock out the expression of HBsAg by targeting the open reading frames, preS1/preS2/S, of the HBV gene in the HCC cell line. Cell culture results showed a dramatic reduction in HBsAg level associated with inhibiting cellular proliferation, IL-6 level, and signal transducer and activator of transcription 3 (STAT3) signalling. Additionally, tumour formation capacity was also blocked, using a similar approach, in animal models (Song et al., 2018). Indeed, *in vitro* and *in vivo* pre-clinical studies showed a solid contribution of the CRISPR/Cas9 modality in producing remarkable eradication of HBV infection by destroying the infected hepatocyte, thus providing a potential promising platform for treating the chronic HBV infection (Ebert et al., 2015a; Ebert et al., 2015b; Ebert and Pellegrini, 2016; Doerflinger et al., 2017).

#### 4.1.3 Human Herpesviruses

Human Herpesviruses belong to a family of *Herpesviridae* within the order Herpesvirales. Eight main herpes viruses can infect humans as a primary host. These include Epstein-Barr virus (EBV), herpes simplex virus (HSV) types 1 and 2, cytomegalovirus (CMV), varicella-zoster virus, Human herpesvirus-6, Human herpesvirus-7, and Kaposi’s sarcoma herpesvirus (Najafi et al., 2022). Like other viruses, herpesviruses can establish latent infection, thus enabling the virus to stay in the infected host for life and reinfect the host periodically during the reactivation and replication period. The current anti-viral treatments cannot eliminate these viruses from the host cells as their effect is limited to the active stage of the virus, thus leaving the dormant viruses unaffected (van Diemen et al., 2016). This viral phenomenon prompted the investigation of an alternative modality, such as gene-editing technologies, to fight against viral infection and ensure complete eradication of the inert viral elements.

##### 4.1.3.1 Epstein-Barr Virus

EBV is the causative organism of oropharyngeal infectious mononucleosis. EBV can also be associated with different cancers, including Hodgkin’s disease, Burkitt’s lymphoma, nasopharyngeal carcinoma, and post-transplant lymphoma (van Diemen et al., 2016). Efficient genomic editing of EBV in human epithelial cell lines, including human nasopharyngeal carcinomas cells, using the CRISPR/Cas9 system was first demonstrated by Yuen et al. (2015). In this study, CRISPR/Cas9 was designed to target a specific sequence in the promoter region of BART (BamHI A rightward transcript) that derives the expression of the virus microRNA. Furthermore, complete eradication of EBV particles from the latently infected human tumour cells and inhibit active viral replications were achieved using the CRISPR/Cas9 strategy to target essential viral genes. Consequently, CRISPR/Cas9 system was proposed as a therapeutic and prophylactic approach to targeting herpesviruses genomes (Wang and Quake, 2014; van Diemen et al., 2016).

In the primary human B cells, CRISPR/Cas9 showed proof-of-principle of gene editing *via* efficient targeting of CDKN2A, which is responsible for the EBV replication (Akidil et al., 2021). The development of nasopharyngeal carcinoma is strongly associated with infection by EBV. Nasopharyngeal carcinoma (CNE-2 cell lines) cellular growth is primarily mediated through overexpression of LMP1 (latent membrane protein 1). Thus, downregulation of LMP1 expression by the CRISPR/Cas9 tool significantly suppressed cancer cell proliferation. Moreover, loss of LMP1 blocked EBV propagation in CNE-2 cancer cells (Huo and Hu, 2019). Furthermore, CRISPR/Cas9 gene-editing showed efficient targeting of several genes (ORF50, ORF72, and ORF73) essential in the reactivation or latency development of the virus in the host cells. Targeting these genes resulted in a deleterious reduction in the latent load of EBV in human Burkitt lymphoma cells in three different infection models (Foreman et al., 2021).

##### 4.1.3.2 Herpes Simplex Virus

HSV is classified into HSV type1, associated with oral cold sores, and HSV type 2, which causes genital herpes. Both are widely spread and highly contiguous DNA viruses which can generate the lytic and lifetime-latent stages of infection (Whitley and Roizman, 2001). CRISPR/Cas9 modality using multiple gRNAs showed a destructive effect of HSV-1 viral genetic elements essential for productive and latent infections from the infected human cells (van Diemen et al., 2016; Khodadad et al., 2020). Additionally, a plasmid-expressed CRISPR/Cas9 system was designed to target UL52 and UL29 genes of HSV-1 in Vero cells. This construct displayed complete elimination of HSV-1 infection without notable cytotoxic effects (Karpov et al., 2019). Several *in vitro* and *in vivo* investigations have reported the effectiveness of the CRISPR/Cas9 system in the editing of the HSV-1 genome and the suppression of the significant viral genes without introducing off-target effects (Russell et al., 2015; Lin et al., 2016; Xu et al., 2016). Numerous reports have demonstrated the ability of the CRISPR/Cas9 system to eradicate HSV-1 latent viral infection by producing loss of function indel mutation in the related proteins of the quiescent HSV-1 genomes (Wang et al., 2005; Ferenczy and DeLuca, 2011; van Diemen et al., 2016; Horlbeck et al., 2016; Oh et al., 2019).

One of the severe complications of HSV-1 latent infection is blindness due to the persistence of the virus reservoirs in the trigeminal ganglia. A recent *in vivo* study conducted by Yin et al.; described the use of a novel Cas9/HELP (designated HSV-1-erasing lentiviral particles) constructs targeting HSV-1 genomes and blocking its replication, thus preventing the development of herpetic stromal keratitis (HSK). This construct showed efficient abolishing of the viral reservoirs in the trigeminal ganglia and cornea without inducing off-target effects, thus proposing the HELP construct as a promising therapeutic tool in HSK refractory patients (Yin et al., 2021). Likewise, infecting the human corneal epithelial cells (HCECs) with CRISPR/Cas9 system targeting NECTIN-1 expression caused attenuation of HSV infection, thus providing another novel modality for HSK treatment (Li et al., 2022). Similar encouraging results were obtained regarding HSV-2; CRISPR/Cas9 system was used to produce a deletion mutation of the two strains of HSV-2, RL1 (Repeat Long element 1) and/or LAT (Latency-associated Transcript) genes. Compared to the wild type, The infected mice with the two mutated strains demonstrated a lower pathogenic phenotype during both phases of infections, acute and latent, and a more competent immune system (Liu et al., 2020).

##### 4.1.3.3 Cytomegalovirus

The clinical presentation of CMV infection ranges from symptomatic or mild harmless symptoms such as skin rash to long-term mental problems involving neurological and behavioural manifestations (Kirby, 2016). Most of the CMV-infected women during pregnancy will be asymptomatic. Yet, CMV infection in the neonates is associated with substantial congenital abnormalities, including chorioretinitis, neurodevelopmental delay, mental or motor impairment, and microcephaly (Hughes and Gyamfi-Bannerman, 2016). The CRISPR/Cas9 system was also reported to have effectively abolished CMV infection by targeting essential genes for viral production and replication and, therefore, eliminating the infectious elements from the human cells (van Diemen et al., 2016). Furthermore, CRISPR/Cas9-mediated genomic engineering technology was employed to dissect the role of the CMV genome play during infection. Homologous recombination (HR) and Non-homologous end-joining (NHEJ)-based approaches were used to produce powerful mutation in the CMV genome with maximum competencies of 42% and 81%, respectively. This method provided a blueprint for studying the mutational analysis of the CMV genome and assisted in understanding the biological functions and the pathogenicity of the CMV genome (King and Munger, 2019). Expression of the immediate-early (IE) genes contributes to the persistence of the CMV latent infection and supports viral replication and reactivation. CRISPR/Cas9 system incorporated with specific single-guide RNAs (sgRNAs) and lentivirus was constructed to target the IE region of the CMV genome. The introduction of CRISPR/Cas9/sgRNA lentiviral constructs efficiently inhibited the viral gene expression in fibroblasts and viral DNA synthesis and reactivation in the THP-1 monocytic cell line (Xiao et al., 2020).

#### 4.1.4 Human Papilloma Virus

HPVs are double-stranded DNA viruses that belong to a family of Papovaviridae, including approximately 150 HPV types recognized so far. HPV can be classified into low-risk groups causing genital warts (like penis, anus, and vulva) and high-risk groups causing the various type of cancers, most commonly female cervical cancers (Baghi et al., 2017). HPV-16 and HPV-18 are the most investigated types. They are highly infectious, causing sexually transmitted diseases associated with cervical cancer development in about 50% and 20% of all cases globally, respectively (Tommasino, 2014). In addition to cervical cancers, HPV is also engaged in causing different types of cancers, including anogenital cancer and warts and head and neck cancers. Like other viruses, latency and dormancy account for the main bottleneck making it extremely difficult to eliminate a viral genome from the infected cells and achieve a satisfactory cure (Chen et al., 2018; Lee, 2019). Numerous studies have been conducted to investigate the role of the CRISPR/Cas9 system in fighting against the HPV genome (Tommasino, 2014; Zhen et al., 2016; Zhen and Li, 2017; Aghamiri et al., 2020; Gao et al., 2021; Inturi and Jemth, 2021; Najafi et al., 2022). Two HPV viral genes were identified as oncogenes that exhibit fundamental roles in deriving malignant cellular transformation, E6 and E7, specifically cervical carcinomas. Therefore, targeting the expression of E6 and E7 is considered a promising tool in treating cervical cancer cases (Tommasino, 2014). *In vitro* and *in vivo* investigations have reported the engagement of the CRISPR/Cas9 system in the downregulation of HPV oncogenes, therefore, promoting cancer cells apoptosis and enhancing their sensitivity to the radiation treatment (Najafi et al., 2022). The antiviral effect of the CRISPR/Cas9 system was further authenticated against E6 and E7 oncogenes of HPV16, the most prevalent inducers of cervical cancers. Both *in vitro* and *in vivo* showed that a combination strategy of the chemotherapeutic agent cisplatin and CRISPR/Cas9 system produced a synergetic effect in inducing cervical cancer cells apoptosis and metastasis suppression (Zhen et al., 2016).

Furthermore, CRISPR/Cas9 system was found to trigger cellular senescence in the Hela cells immortalized with HPV-18 by knocking down the expression of E6/E7 oncogenes. E6/E7-inhibited Hela cells manifested with increased β-galactosidase level, loss of lamin B1, and activation of p53/p21 and pRb/p21pathway (Inturi and Jemth, 2021). The ability of the CRISPR/Cas9 system was further investigated in cervical cancer/pre-cancer cell lines and animal models by Gao et al. (2021). In this study, transfection of the S12 cell line (low-grade cervical lesion) with HPV16 E7 targeted CRISPR/Cas9 resulted in significant suppression of cell growth, colony formation, and apoptotic capacity. Moreover, introducing the CRISPR/Cas9 system into the transgenic mice model of HPV-induced cervical carcinogenesis revealed similar encouraging effects. CRISPR/Cas9 treatment-induced mutation of the E7 gene increased the expression of RB, E2F1, and CDK2, yielding a less aggressive phenotype (Gao et al., 2021). CRISPR/Cas9 system has shown encouraging results in cervical cancer targeted therapy. However, many obstacles involving off-target effects, immunogenic reactions, renal elimination, degradation of the guide RNA (gRNA) nuclease, delivery, and transportation system limited the application of this robust technology (Aghamiri et al., 2020; Noroozi et al., 2022). Therefore, further validation of this technology is required to ensure safety and feasibility (Zhen and Li, 2017).

In summary, the findings of these simulations *in vitro* and *in vivo* models propose the CRISPR/Cas9 system as a powerful, talented platform to eradicate different types of viral infections. Nonetheless, further investigations are needed to understand better the mechanism of CRISPR/Cas9 in fighting against these viruses’ genomes.

### 4.2 Other Non-Viral Infectious Pathogens Targeted by CRISPR Technology

CRISPR/Cas9 is an elegant, reprogrammed tool that assists in fighting against bacterial pathogens. CRISPR interference modality (CRISPRi) was successfully utilized to dampen the transcriptional activity of numerous bacteria genes related to M. tuberculosis, Pseudomonas aeruginosa, Klebsiella pneumonia, and Escherichia coli (Doerflinger et al., 2017). Moreover, by applying the technique of CRISPR to study malaria parasitic infection, Hammond et al. were able to edit three different genes in the female mosquito of malaria (Anopheles gambiae), leading to sterile females and failure of reproduction and transmission of the infection (Ghorbal et al., 2014; Hammond et al., 2016). The anti-parasitic intervention of CRISPR has expanded to overcome other parasitic diseases such as toxoplasmosis caused by Toxoplasma gondii, leishmaniasis (Leishmania), and Trypanosomiasis/sleeping sickness (Trypanosoma) (Doerflinger et al., 2017).

## 5 COVID-19 Pandemic Tragedy, Emergence, and Spread

Coronavirus disease 2019 (COVID-19) is a rapidly emerging infectious disease that was declared a pandemic on 13 March 2020. According to the WHO situation report, 19,462,112 established cases of COVID-19 were reported globally by August 9^th^, 2020, leading to 722,285 mortalities. COVID-19 is triggered by the severe acute respiratory syndrome coronavirus-2 (SARS-CoV-2) (Coronaviridae Study Group of the International Committee on Taxonomy of V., 2020). This was transmitted through breathing, aerosol particles, and direct touching of contaminated abiotic surfaces. This virus has 93.3% similarity with the genome of bat coronavirus (denoted RmYN02) (Zhou H et al., 2020) and 79.5% similarity when compared to SARS-CoV (Zhou P et al., 2020). Unlike SARS-CoV, SARS-CoV-2 has a faster frequency of human-to-human transmission. SARS-CoV-2 targets the respiratory tract causing damage and lysis of the lining epithelial cells. This resulted in the profound release of different inflammatory mediators and a severe immunological reaction (Song et al., 2022). This pandemic has prompted the scientific community to conduct considerable research to generate diagnostic tests, effective and safe medications, and vaccines to curb the spread of the virus and battle COVID-19. Hence, genome editing technologies, such as CRISPR present novel opportunities to meet this aim. Indeed, genome editing tools can target viral RNA and can be specifically engineered to target the COVID-19 RNA genome to restrict virus progression and transmission within the population (De Masi et al., 2020).

### 5.1 COVID-19 Treatment and Prevention Landscape

Since the beginning of the COVID-19 pandemic in China in Dec-2019, millions of populations have been affected globally, indicating the high contagious capacity associated with SARS-CoV-2. As producing an effective vaccine for this deadly disease is time-consuming, investigating the efficacy of some available drugs would provide a faster and potentially promising approach. Therefore, tremendous randomized controlled trials (RCTs) were conducted to study the effectiveness of already approved medications in treating COVID-19 disease. In a comprehensive, well-conducted study, JAK-inhibitors (Baricitinib) and IL-6 receptor antagonists (tocilizumab and sarilumab) were both drugs commonly used in inflammatory disease and suggested as therapeutic modalities for COVID-19. Administration of IL-6 receptor antagonists and JAK-inhibitors to hospitalized ill COVID -19 patients displayed good clinical outcomes and improved patients’ medical responses and survival (Tandon et al., 2022; Villaescusa et al., 2022).

Furthermore, a metanalysis of other RCTs declared that using systemic corticosteroids (prominent anti-inflammatory drugs) resulted in a decline in the mortality rate in ICU ill COVID-19 patients (Boppana et al., 2021). For COVID-19 patients with stable conditions, adding Mefenamic acid (non-steroidal anti-inflammatory) to the standard care regimen on ambulatory COVID-19 patients was associated with a significant reduction in symptoms and shortage of the recovery period in comparison to the control group. As Mefenamic acid could exhibit antiviral effects, it was suggested to add this medicine to other anti-viral drugs (remdesivir, molnupiravir, or favipiravir) to treat COVID-19 stable patients (Guzman-Esquivel et al., 2022). Another anti-inflammatory modulator CD24Fc was verified in a phase III randomized trial that showed remarkable suppression of systemic inflammation in the infected tested patients compared to the placebo ones. By regulating the response of multiple inflammatory pathways and inducing balanced homeostasis, CD24Fc was proposed as a promising therapeutic agent for COVID-19 patients (Song et al., 2022).

On the contrary, other repurposed drugs such as hydroxychloroquine (for Malaria), lopinavir/ritonavir (for HIV), and ivermectin (Antiparasitic) didn’t demonstrate any clinical effectiveness against COVID-19. Additionally, mild to moderate gastrointestinal and cardiovascular adverse effects were reported by (Chiu et al., 2022). Moreover, using the combination therapy of P2Y12 (platelets inhibitors) and heparin didn’t improve clinical outcomes compared to patients who received heparin only (Berger et al., 2022). The war hasn’t been over yet; passive immunization using hyperimmune intravenous immunoglobulin (hIVIG) against SARS-CoV-2 or COVID-19 convalescent plasma (CCP) didn’t show any beneficial clinical effects in the examined group as compared to the placebo group. Also, the safety level of using the hIVIG approach was varied among the individuals (2022; Troxel et al., 2022; Ray et al., 2022).

Numerous reports have been involved in vaccine development, and currently, many vaccines have been FDA approved for human use. These vaccines include two mRNA-based vaccines, BNT162b2 (Pfizer-BioNTech) and mRNA-1273 (Moderna), a chimpanzee adenovirus-vectored vaccine, ChAdOx1 nCoV-19 (Oxford-AstraZeneca), a non-replicating viral vector vaccine, Sputnik V (Gamaleya Research Institute), and two inactivated vaccines, CoronaVac (Sinovac Biotech) and BBIBP-CorV (Sinopharm) (Tanne, 2020; Crasto, 2021; COVID, N.J.I., 2021; Goepfert et al., 2021). This follows breakneck efforts by many researchers to generate more specific and highly effective vaccines to achieve complete eradication of this pandemic infection. Prominent countries leading vaccine development include Russia, the USA, China, India, and the UK. One of the most advanced vaccines in nonrandomized phase 1/2 studies is a vaccine developed in Russia by the Gamaleya Research Institute of Epidemiology and Microbiology (Logunov et al., 2020). Mechanically, the glycoprotein S (spike) protein and ACE2 receptors interact and enable the entrance of the SARS-CoV-2 virus into the cells. Therefore, researchers have proven that blocking this interaction reduces the internalization and replication of the virus (Datta et al., 2020). The unique feature of this vaccine is that it comprises two heterologous vectors, both of which convey the gene for the SARS-CoV-2 full-length glycoprotein S, recombinant adenovirus type 26 (rAd26), and recombinant adenovirus type 5 (rAd5). Using two different vectors that carry the same gene enhances the body’s capability to stimulate the immune system to produce antibodies in the long term. However, this vaccine and others currently in phase III need to be further investigated to prove their effectiveness in preventing COVID-19 and await appropriate agencies’ approval for human use (Logunov et al., 2020; Goepfert et al., 2021; Sridhar et al., 2022).

### 5.2 Engagement of the Genome-Editing CRISPR Technology in Battling COVID19

The selectivity and sensitivity of the diagnostic tool are required to track the disease and constrain its transmission. Two diagnostic CRISPR-based tools have been used in the detection of viral and bacterial nucleic acid, DETECTOR (for RNA- using Cas12a) and SHERLOCK (for DNA-using Cas13a) (Gootenberg et al., 2017). These modalities are fast (1-2 h), relatively low-cost, and highly sensitive Fields (Zhang et al., 2020; Kellner et al., 2020). After amplifying the organism’s nucleic acid, the DNA product is mixed with the CRISPR-Cas system that detects and cuts the targeted sequence. After cleaving the series of interest, Cas12a and Cas13a begin to cleave other non-specific nucleic acids in the solution. Then both enzymes, Cas12a and Cas13a, will cleave the reporter quenched sequence leading to fluorescent emission that can be quantified. These tests were proposed to diagnose several viral infections caused by Ebola, Zika, and Dengue, and recently for the detection of SARS-CoV-2 (Katalani et al., 2020; Broughton et al., 2020; Guo et al., 2020; Ding et al., 2020; Nguyen et al., 2020; Binnie et al., 2021).

A recent diagnostic tool is based on CRISPR technology using an easy-readout and sensitive enhanced (ERASE) strip. This tool provided a rapid, simple, highly sensitive and specific assay to detect SARS-CoV-2 positive cases (Li et al., 2021). Furthermore, another diagnostic modality combining the CRISPR system and RT-PCR (CRISPR-augmented RT-PCR) assay showed high efficacy and accuracy in detecting and quantifying the virus RNA from the plasma samples rather than the traditional test using nasal/nasopharyngeal swab. While the conventional RT-qPCR shows less sensitivity for blood samples (~ 44%), the CRISPR-augmented RT-PCR assay revealed 91.2% sensitivity and 99.2% specificity. Also, this approach can accurately detect positive COVID-19 cases with negative nasal swabs. Thus, highlighting the validity of the CRISPR-augmented RT-PCR strategy in providing a precise plausible diagnostic tool for SARS-CoV-2 infection and assessing its severity, progression, and clearance (Huang et al., 2021).

The CRISPR-based system also presented a valuable contribution to treating COVID-19 disease. An elegant therapeutic modality named PAC-MAN (prophylactic antiviral CRISPR in human cells) for targeting SARS-CoV-2 was proposed. This modality is based on aerosol delivery of a novel CRISPR-Cas13 system integrated with 6 CRISPR guide RNAs (crRNAs) targeted to conserved sequences of SARS-CoV-2. This should be approached with the assistance of the Adeno-associated virus to increase the binding affinity of this modality to the epithelial cells in the lungs. This modality showed a 90% reduction in the virus load (Yan et al., 2018; Nguyen et al., 2020; Abbott et al., 2020; Binnie et al., 2021). The PAC-MAN strategy is superior to conventional vaccination methods that rely on the activation of the immune system to produce neutralizing antibodies in response to viral particles such as peptides. This approach increases the chance of virus resistance secondary to mutation of these viral peptides as a natural defence mechanism. On the other hand, PAC-MAN prevents the virus escape from the immune system by attacking the conserved regions and thus blocking further mutations (Zhu et al., 2020). Moreover, Cas13 can attack many regions in the virus, thus enhancing virus clearance. Although several delivery approaches for CRISPR-Cas13 have been suggested, such as lipid nanoparticles and high-level extended duration gene expression system (HEDGES), finding a safe and efficient *in vivo* delivery vehicle remains the major roadblock in the PAC-MAN strategy (Abbott et al., 2020). Therefore, further investigations to confirm the efficacy and safety of this strategy should follow.

ABACAS is another innovation to fight against SARS-CoV-2 infected cells specifically. This technology relies on the fusion of CRISPR-Cas13 to the specific antibody against the S protein on the SARS-CoV-2 virus permitting internalization of this construction and the viral RNA selectively into the infected cells only. Thus, resulting in suppression of the virus and the elimination of unintended effects on off-tissue (Yuan et al., 2020). Unlike the PAC-MAN system, we don’t require a delivery vehicle as the construct allows the CRISPR entry into the cells. While this innovative approach is considered a good treatment for COVID-19, comprehensive investigations of this antiviral ability and the underlying mechanisms and biological processes are indispensable.

## 6 Uses of CRISPR/Cas9 System in Clinical Trials

A small *ex-vivo* model was done on three patients with advanced cancer. T-cells were harvested and subjected to CRISPR engineering externally and then re-introduced to the patients. However, all three patients showed sufficient expression of the modified T-cells; nine months after the procedure, two were alive, and the third one had died (Kalos et al., 2011). The first *in vivo* human trial for using the CRISPR system started in early 2020. In this trial, an engineered adenovirus-CRISPR-based system will be delivered *via* subretinal injection into one eye of a patient with congenital retinal disease. This technology is aimed to edit a single base mutation which is the underlying cause of this medical condition. The outcomes of this study are still awaiting (First CRISPR Therapy Dosed 2020).

## 7 Conclusion and Future Perspectives

This review presented a brief history of the most common genome editing modalities focusing on the CRISPR-Cas9 tool and its widespread engagement in managing viral and non-viral infections. We also briefly discussed the application areas of CRISPR technology in vaccine establishment and the recently conducted clinical trials using the CRISPR system in treating different medical conditions. Undoubtedly, CRISPR has fueled the scientific and medical fields with unprecedented tools and strategies, yet the off-target effects and the mode of delivery remain a concern. Therefore, before moving to clinics, improving the design of sgRNA and the delivery vehicle to increase selectivity and efficacy and avoid unwanted effects is warranted. After that, we explained the current COVID-19 pandemic and the critical developments in diagnosis, treatment, and vaccine advancement to fight SARS-CoV-2 infection. Finally, we elucidated the novel and promising contribution of the CRISPR system as an antiviral modality in fighting against COVID-19 disease. Looking forward, COVID-19 provides an opportunity to evaluate the future potential use of CRISPR-based systems as therapeutic and diagnostic tools in combating viral infections.

The road is not free of challenges. The war against COVID-19 hasn’t ended yet due to multiple recent variants as this virus is continuously acquiring new mutations causing serious therapeutic obstacles and limitations. The mutation is a natural protective mechanism exhibited by the viruses to help them survive and give them resistance to antiviral agents. Thus, the generation of many mutant strains enables numerous human viruses to escape the immune system or antiviral modalities (i.e., virus escape phenomena) and further jump between different species causing pandemic infection (Lin et al., 2021). This contains the medical and scientific communities in an ongoing battle to continuously amend the antiviral modalities, including drugs and vaccines (Yan et al., 2018; Nguyen et al., 2020; Abbott et al., 2020; Binnie et al., 2021). This review will expand our vision and raise the hope of using the CRISPR system along with other advanced modalities to shed light on finding unrivalled techniques that can overcome all challenges accompanying COVID-19 disease. The rallying of global efforts in this area will lead to curbing the spread of this pandemic illness and enable the application of gene editing technologies to find a solid solution to other diseases of global importance.

## Author Contributions

Conceptualization and writing of original draft preparation: YH, AT, and NA. Formal analysis and writing: JS, MB, NM, OA. and WA Reviewing, writing, and editing: AS Resources and visualization: NA, RA. AA, AMA and OA Supervision: Y.H. Project administration: YH and OA. All authors have read and agreed to the published version of the manuscript.

## Conflict of Interest

The authors declare that the research was conducted in the absence of any commercial or financial relationships that could be construed as a potential conflict of interest.

## Publisher’s Note

All claims expressed in this article are solely those of the authors and do not necessarily represent those of their affiliated organizations, or those of the publisher, the editors and the reviewers. Any product that may be evaluated in this article, or claim that may be made by its manufacturer, is not guaranteed or endorsed by the publisher.

## Abbreviations

AAV, adeno-associated virus; AIDS, Acquired immunodeficiency syndrome; COVID-19, Coronavirus disease 19; CRISPR, clustered regularly interspaced short palindromic repeats; DMD, Duchenne Muscular Dystrophy; DSBs, DNA double-strand breaks; ES, embryonic stem cells; gRNA, guide RNAs; HIV, human immunodeficiency virus; HDR, homology directed repair; iPS, induced pluripotent stem cells; MERS, Middle East respiratory syndrome; NHEJ, non-homology end joining; PAM, protospacer adjacent motif; RVD, repeat variable di residue; TALENs, transcription activator-like effector nucleases; ZFNs, Zinc-finger nucleases.
